# Rv0100, a proposed acyl carrier protein in *Mycobacterium tuberculosis*: expression, purification and crystallization. Corrigendum

**DOI:** 10.1107/S2053230X2000271X

**Published:** 2020-04-02

**Authors:** Jasper Marc G. Bondoc, Hiten J. Gutka, Mashal M. Almutairi, Ryan Patwell, Maxwell W. Rutter, Nina M. Wolf, Ram Samudrala, Shahila Mehboob, Alexey Dementiev, Cele Abad-Zapatero, Farahnaz Movahedzadeh

**Affiliations:** a Institute for Tuberculosis Research, College of Pharmacy, University of Illinois at Chicago, 833 South Wood Street, Chicago, IL 60612, USA; b Oncobiologics Inc., 7 Clarke Drive, Cranbury, NJ 08512, USA; c Department of Pharmacology and Toxicology, College of Pharmacy, King Saud University, Riyadh 12371, Saudi Arabia; d Vaccines and Biologics Research Unit, College of Pharmacy, King Saud University, Riyadh 12371, Saudi Arabia; e Department of Pediatrics and Molecular Virology and Microbiology, National School of Tropical Medicine, Baylor College of Medicine, 1 Baylor Plaza, Houston, TX 77030, USA; f Department of Psychiatry, University of Illinois at Chicago, 1601 West Taylor Street, Room 425, Chicago, IL 60612, USA; g Hollingbery and Son Hops Inc., 302 North First Avenue, Yakima, WA 98907, USA; h Department of Biomedical Informatics, Jacobs School of Medicine and Biomedical Sciences, State University of New York (SUNY), University at Buffalo, 77 Goodell Street, Buffalo, NY 14203, USA; i NovaScan Inc., 950 N. 12th Street, Milwaukee,, WI 53233, USA; j Department of Pharmaceutical Sciences, College of Pharmacy, University of Illinois at Chicago, 833 South Wood Street, Chicago, IL 60612, USA; k Structural Biology Center, Advanced Photon Source, Argonne National Laboratory, 9700 S Cass Ave, Lemont, IL 60439, USA; l Center for Biomolecular Sciences, University of Illinois at Chicago, Chicago, IL 60612, USA

**Keywords:** acyl carrier protein, *Mycobacterium tuberculosis*, *Mycobacterium smegmatis*, Rv0100, corrigendum

## Abstract

The true identity of the protein found in the crystals reported by Bondoc *et al.* [(2019), *Acta Cryst.* F**75**, 646–651] is given.

## Abstract 2

A correction is made to the article by Bondoc *et al.* [(2019). *Acta Cryst.* F**75**, 646–651].

As described in the article by Bondoc *et al.* (2019 ▸), attempts to solve the crystal structure (2 monomers per a.u. in the *H*3 cell), using existing structural homologs of Rv0100 *Mycobacterium tuberculosis* from the Protein Data Bank failed. Structural models obtained from the amino-acid sequence using the I-THASSER protein prediction server also failed. Refinement of tentative solutions obtained by *MOLREP* and *PHASER* stalled at *R*_w_ = 0.44, *R*_f_ = 0.51, even when assuming a lower symmetry *P*1 lattice with nearly *H*3 pseudo-symmetry. Detailed examination of the PDB entries related to proteins from *Mycobacterium smegmatis*, the organism that was used as an expression strain, revealed the existence of entry 3gwm corresponding to the crystal structure of the holo-[acyl-carrier-protein] synthase (ACPS). This structure was released in 2010 (Poulsen, C., Wilmanns, M. & Song, Y. H., unpublished work). The crystallographic parameters for this deposition (space group *H*3, *a* = 67.45, *b* = 67.45, *c* = 86.06 Å, α = β = 90, γ = 120°) strongly suggested that it was the same protein as the one we had crystallized. Using the protein structure from entry 3gwm, a molecular replacement solution was readily found and was further refined to *R*_w_ = 0.19, *R*_f_ = 0.25 following standard protocols using the previously reported crystallographic data to 2.0 Å resolution. The amino-acid sequence of the refined protein was fully consistent with the sequence in the ACPS entry, and significant structural differences were found only in the proximity of the three SO_4_ anions bound in 3gwm but not in the serendipitously found structure, since there was no sulfate in our crystallization media. The r.m.s.d. between 128 equivalent Cα carbons in the two structures was 0.58 Å (mean deviation 0.33 Å).

We hypothesize that our expression in the *M. smegmatis* strain resulted in the co-purification of ACPS protein from *M. smegmatis*, as a minor contaminant. This is consistent with the fact that only two very small (10–20 µm) crystals were observed in the crystallization droplet and it took about nine months for these crystals to appear. We are certain that the predominant purified protein is Rv0100 from *M. tuberculosis* confirmed through both mass spectrometry and the fact that the histidine tag was used to isolate the protein. We are confident that the ACPS protein (MW ≃ 14 000 Da) was a minor contaminant that co-purified alongside Rv0100 whose molecular weight is significantly different (11 122 Da).
